# Old drugs, old problems: where do we stand in prediction of rheumatoid arthritis responsiveness to methotrexate and other synthetic DMARDs?

**DOI:** 10.1186/1741-7015-11-17

**Published:** 2013-01-23

**Authors:** Vasco Crispim Romão, Helena Canhão, João Eurico Fonseca

**Affiliations:** 1 Rheumatology Research Unit, Instituto de Medicina Molecular - Faculdade de Medicina da Universidade de Lisboa, Edifício Egas Moniz - Av. Prof. Egas Moniz, Lisboa 1649-028, Portugal; 2 Rheumatology Department, Lisbon Academic Medical Centre, Av. Prof. Egas Moniz, Lisboa 1649-028, Portugal

**Keywords:** Predictors of response, Rheumatoid arthritis, Synthetic DMARDs, Methotrexate

## Abstract

Methotrexate (MTX) is the central drug in the management of rheumatoid arthritis (RA) and other immune mediated inflammatory diseases. It is widely used either in monotherapy or in association with other synthetic and biologic disease modifying anti-rheumatic drugs (DMARDs). Although comprehensive clinical experience exists for MTX and synthetic DMARDs, to date it has not been possible to preview correctly whether or not a patient will respond to treatment with these drugs. Predicting response to MTX and other DMARDs would allow the selection of patients based on their likelihood of response, thus enabling individualized therapy and avoiding unnecessary adverse effects and elevated costs. However, studies analyzing this issue have struggled to obtain consistent, replicable results and no factor has yet been recognized to individually distinguish responders from nonresponders at treatment start. Variables possibly influencing drug effectiveness may be disease-, patient- or treatment-related, clinical or biological (genetic and nongenetic). In this review we summarize current evidence on predictors of response to MTX and other synthetic DMARDs, discuss possible causes for the heterogeneity observed and address its translation into daily clinical practice.

## Introduction

Methotrexate (MTX) is the anchor disease modifying anti-rheumatic drug (DMARD) in the management of rheumatoid arthritis (RA) and other immune mediated chronic inflammatory disorders. In RA, it is the most commonly used DMARD and the first one to be chosen [1,2] due to its efficacy, safety and cost, ultimately translated by the best drug retention rate among DMARDs [3-6]. It is the gold standard in the management of RA and can be prescribed in monotherapy or in combination with other synthetic or biological agents [7-9]. Multinational recommendations have been issued for the use of MTX in RA management [10] and are summarized in Table 1. However, MTX is not effective or induces significant adverse events in a considerable number of patients [11] who are forced to discontinue it and switch to another DMARD regimen, generally with equally heterogeneous responses [5].

**Table 1:** Multinational recommendations for the use of methotrexate in RA.

Recommendation		Level of evidence	Grade of recommendation	Selected references
1	The work-up for patients starting methotrexate should include clinical assessment of risk factors for methotrexate toxicity (including alcohol intake), patient education, AST, ALT, albumin, CBC, creatinine, chest × ray (obtained within the previous year); consider serology for HIV, hepatitis B/C, blood fasting glucose, lipid profile and pregnancy test.	4	C	[39,231-237]
2	Oral methotrexate should be started at 10 to 15 mg/week, with escalation of 5 mg every 2 to 4 weeks up to 20 to 30 mg/week, depending on clinical response and tolerability; parenteral administration should be considered in the case of inadequate clinical response or intolerance.	2b	B	[238-242]
3	Prescription of at least 5 mg folic acid per week with methotrexate therapy is strongly recommended.	1a-	A	[207,243-245]
4	When starting methotrexate or increasing the dose, ALT with or without AST, creatinine and CBC should be performed every 1 to 1.5 months until a stable dose is reached and every 1 to 3 months thereafter; clinical assessment for side effects and risk factors should be performed at each visit.	4	C	[39,233,246-250]
5	Methotrexate should be stopped if there is a confirmed increase in ALT/AST greater than three times the ULN, but may be reinstituted at a lower dose following normalization. If the ALT/AST levels are persistently elevated up to three times the ULN, the dose of methotrexate should be adjusted; diagnostic procedures should be considered in the case of persistently elevated ALT/AST more than three times the ULN after discontinuation.	2b	C	[237,250-252]
6	Based on its acceptable safety profile, methotrexate is appropriate for long-term use.	2b	B	[253-258]
7	In DMARD-naïve patients the balance of the efficacy/toxicity favors methotrexate monotherapy over combination with other conventional DMARDs; methotrexate should be considered as the anchor for combination therapy when methotrexate monotherapy does not achieve disease control.	1a-	A	[7,57,259-263]
8	Methotrexate can be safely continued in the perioperative period in RA patients undergoing elective orthopedic surgery.	1b	B	[264,265]
9	Methotrexate should not be used for at least three months before planned pregnancy for men and women and should not be used during pregnancy or breast feeding	4	C	[266-268]

Adapted by permission from BMJ Publishing Group Limited. [*Ann Rheum Dis*, Visser K *et al*. 2009, **68:**1086-1093]. Evidence-based recommendations for the use of methotrexate in daily clinical care of rheumatic disorders, issued by 751 rheumatologists from 17 countries through systematic review of the literature, discussion and voting. Levels of evidence and grades of recommendation were determined according to those established by the Oxford Centre for Evidence Based Medicine [269], as follows: level 1a - systematic review (SR; with homogeneity) of randomized clinical trials (RCTs); level 1b - individual RCT (with narrow confidence interval); level 1c - all or none; level 2a - SR (with homogeneity) of cohort studies; level 2b - individual cohort study (including low quality RCT); level 2c - 'outcomes' research, ecological studies; level 3a - SR (with homogeneity) of case-control studies; level 3b - individual case-control study; level 4 - case-series (and poor quality cohort and case-control studies); level 5 - expert opinion without explicit critical appraisal or based on physiology, bench research or 'first principles'; a minus sign (-) can be added to a particular level, to denote that it fails to provide a conclusive answer because of either a single result with a wide confidence interval or a SR with troublesome heterogeneity. Grade of recommendation A - consistent level 1 studies; grade of recommendation B - consistent level 2 or 3 studies or extrapolations from level 1 studies; grade of recommendation C - level 4 studies or extrapolations from level 2 or 3 studies; grade of recommendation D - level 5 evidence or troublingly inconsistent or inconclusive studies of any level. ALT, alanine aminotransferase; AST, aspartate aminotransferase; CBC, complete blood count; DMARD, disease-modifying antirheumatic drug; RA, rheumatoid arthritis; SD, standard deviation; ULN, upper limit of normal.

Being able to predict response to first-line DMARDs, has been one of the main challenges in RA management for over two decades [12] and it is a good example of the increasingly appealing concept of personalized therapy, that is, choosing the drug of most benefit for a particular patient. This would be of tremendous benefit in several ways. By identifying patients less prone to respond it would avoid needless exposure to potentially toxic drugs and the waste of precious time to achieve disease control, a crucial endpoint to prevent development of structural damage [13]. Likely responders would be maintained with the most appropriate DMARD with more certainty, obviating an early, possibly unnecessary, switch to other potentially less effective DMARDs or to more costly biologicals. Theoretically, this would allow physicians to quit the current trial-and-error approach and adopt solid, objective criteria of targeted drug selection, leading to cheaper, quicker, safer and more effective control of the disease.

However, this has proved to be an arduous task and to date there are few clear, reliable, variables that can be used in daily practice to allow prediction of response to MTX or other DMARDs [14-19]. While predictors of poor RA prognosis are well established [20,21], they do not accurately correlate with response to treatment [16]. Furthermore, heterogeneous response is most likely the result of multi-factor interactions and cannot be explained by a single cause-effect mechanism within a certain domain. Those factors that are possibly influencing drug effectiveness can be divided into patient-related (age, gender, ethnicity, comorbidities), disease-related (duration, activity, disability, biomarkers), treatment-related (compliance, dose, previous drugs) and genetic factors [16]. We conducted a literature review to summarize current available data on predictors of response to MTX and other DMARDs (dividing them into clinical factors, nongenetic biomarkers and genetic biomarkers), discuss the causes for the discrepancies reported and critically analyze the possible translation into clinical practice.

## Clinical predictors of response

Several clinical factors have been studied and it has been difficult to reach a consensus on which factors are undoubtedly predictive of response to treatment with MTX and other DMARDs (Table 2).

**Table 2:** Summary of clinical predictors of response to MTX and other DMARDs.

Factors	Predictor of response?	Comments
Gender	Yes, men respond better to MTX	Both in early and established RA; not extendable to other DMARDs
Age	No	Strong evidence showing lack of influence on MTX responsiveness, a few contradicting studies; probably also no influence on other DMARDs
Ethnicity	Uncertain	Despite the theoretical rationale, more data is needed
Smoking	Likely, active smokers respond worse to MTX	Most studies do not analyze it; however, available studies point to worse response in active smokers; not certain as to extension to other DMARDs
Disease duration	Yes, better response in early RA	Early RA has better response than established RA, but no influence of duration in longstanding disease; controversial results due to methodological heterogeneity
Prior DMARD use	Yes, worse response	Previous DMARD use associated with worse response to MTX and other DMARDs; however, not confirmed in some studies; results likely to be affected by several clinical confounders
Disease activity measured by composite scores	Yes, worse response if higher baseline activity	However, frequently not replicable with different scores; unclear which scores are better, likely to depend on response measures used
Disease activity measured by isolated variables	Uncertain	Contradicting findings; unreliable when variables are used separately
Disability	Uncertain, not likely	No association in most studies; inverse relation between HAQ and response in some early RA studies
Pain global assessment	No	Strong evidence showing lack of influence on response to MTX and other DMARDs; a few contradicting studies
Concomitant NSAIDs	Uncertain	Only two studies suggesting higher response to MTX in NSAIDs users; more data needed
Concomitant corticosteroids	Likely, better response	Although most studies fail to analyze it, combined therapy with steroids seems to have better results than DMARD monotherapy
Radiographic scores	No	Extensively shown that baseline radiographic scores do not predict clinical response to any DMARD

Conclusions and comments are based on the findings reported and discussed in the text. DMARDs, disease modifying anti-rheumatic drugs; HAQ, health assessment questionnaire; MTX, methotrexate; NSAIDs, non-steroidal anti-inflammatory drugs; RA, rheumatoid arthritis.

Regarding gender, it seems that men respond better to MTX than women: in the systematic review by Drouin *et al. *[15], the authors found that male gender was associated with a better clinical response to MTX both in early [22] and established RA [23]. Similar conclusions were reached by Anderson *et al*. in a large meta-analysis of randomized controlled trials (RCTs), including 1,435 patients, in terms of achieving American College of Rheumatology (ACR) 20 responses [24], and by Saevarsdottir and colleagues [25], in a population of early RA patients (SWEFOT trial), with a worse European League Against Rheumatism (EULAR) response being observed in women (odds ratio (OR) = 0.50, 95% confidence interval (CI) 0.31 to 0.81). Stranzl *et al*. also found female sex to be an independent predictor of poor response to MTX (OR = 3.3, *P *= 0.009) [26]. In the study by Vázquez *et al. *[27], in early RA patients, male gender was associated with remission after two years of MTX ± gold treatment in the univariate analysis but not in the multivariate analysis. Hider *et al. *[14] found no differences between men and women in response rates to MTX in a prospective study of an early inflammatory polyarthritis cohort and there are also other studies that were not able to identify an influence of gender on MTX response [28]. In spite of some conflicting results, it seems that most of the evidence points in the direction of male gender being a predictor of good response to MTX in both early and established RA. Indeed, in a recently published study [29], a predictive model for 24-month remission was developed for patients with early RA treated in a RCT with MTX ± corticosteroids ± cyclosporine [30]; it was validated in an early RA cohort (ERAN) of patients treated with MTX or other DMARDs [31]. The authors concluded that one of the three variables that predicted remission at 24 months was male gender (OR = 3.14, *P *<0.001). As in this latter study, most of the analyses of response to other DMARDs have been done together with MTX, so their individual effect is difficult to predict. Two publications from the 1990s, a meta-analysis [32] and an observational study [33], comprising a significant number of patients, demonstrated that gender did not influence the response to treatment with sulphasalazine (SSZ), gold and penicillamine. A more recent open label trial showed no influence of gender on whether patients with early RA started on hydroxychloroquine (HCQ) would have to step up therapy to MTX [34]. Other studies have also failed to detect a significant effect of gender on treatment response to DMARDs, other than MTX [28,32,35-37]. Overall, it seems that under the light of current evidence it is not possible to generalize the better response to MTX treatment seen in men to other DMARDs. The possible explanation of the influence of gender on MTX responsiveness, as proposed by Hider *et al. *[16], might be that hormonal factors that influence the pharmacokinetics and pharmacodynamics of each drug contribute to a better or worse response, explaining the apparent discrepancy in the influence of this factor on different DMARDs. Another question could be raised based on the fact that female gender is *per se *predictive of a worse global prognosis in terms of radiographic progression and disability [21,38]: is the female worse response to treatment with MTX contributing to this observation? This is a hypothesis that needs further investigation.

Age does not seem to be a predictive factor of response to MTX or to other DMARDs. Most studies showed a lack of effect of baseline age on clinical response to MTX therapy, including two large meta-analyses [15,39], and, therefore, it does not seem to influence responsiveness [14,23,24,26-28,32,37,40-42]. Despite this strong body of evidence, some studies have contradicting results, such as the SWEFOT trial [25] which showed that older age was associated with a higher likelihood of both EULAR and the clinical disease activity index (CDAI) response to MTX treatment at three to four months (OR = 1.30, 95% CI 1.11 to 1.51) and the study by Ma *et al. *[29], where older patients (>50 years old), on the contrary, were less likely to be in remission at 24 months after the start of MTX ± cyclosporine (OR = 0.97, *P *= 0.014). Thus, despite these two early RA studies, where age seemed to influence response to MTX treatment, although in opposite directions, most studies, including large meta-analyses, showed that age is not a predictor of response to MTX. As to other DMARDs, a single study showed that patients responding to SSZ were younger than non-responders, with no effect of age on the response to penicillamine and gold [33]. All other publications excluded age as an independent predictive marker of response to DMARDs [24,28,34,35,42].

Ethnicity may play a role in predicting response to DMARDs. Genetic differences influencing drug-metabolizing enzymes can contribute to a differential response between ethnic groups [16]. This can have a tremendous impact in either limiting the ability to generalize data from clinical trials to different population groups or choosing the best DMARD for a specific patient based on his/her ancestry. This can be particularly relevant in some European and North American geographical areas, where patients' origins can be very heterogeneous. Helliwell and Ibrahim reported differences in DMARD drug survival, with South Asian patients discontinuing therapy sooner than patients from Northern Europe [43]. Although inefficacy was one of the reasons for drug discontinuation, it was only patient-reported and not objectively measured and it seemed that other factors, such as adverse reactions and different expectations concerning the disease and treatment, may have weighed more than efficacy in the decision to stop treatment. Moreover, some authors found no association between ethnicity and likelihood of response [44,45] and most studies do not analyze its predicting role. Thus, despite the favorable theoretical rationale, ethnicity is currently not a definite predictor of response to MTX and other DMARDs and more data with large populations are needed to clarify its influence on responsiveness.

Smoking has a negative impact on disease outcomes and is associated with higher disease activity [46-49]. In addition to that, smokers seem to respond worse to MTX. Although most studies on treatment response did not analyze this factor, there seems to be a common conclusion in the ones that did: current smokers do respond worse to MTX treatment, at least in early RA. Wessels *et al*. showed that early RA patients who smoke and are rheumatoid factor (RF)-positive have a worse response to MTX monotherapy (OR = 0.1, 95% CI 0.0 to 0.4) [22]. In an early RA cohort, where 873 patients started MTX monotherapy at inclusion, current smoking was independently associated with significantly worse early and late EULAR, disease activity score (DAS) 28 and joint count responses, when adjusted for other clinical, serologic and genetic factors (OR = 0.60, 95% CI 0.39 to 0.94) [50]. Past smokers' responses did not differ from never-smokers' and number of pack-years smoked was not associated with responsiveness [50]. Data from the SWEFOT trial confirmed these findings on a similar population and current smoking was the strongest predictor of achieving a poor response (OR = 0.35, 95% CI 0.20 to 0.63), according to all response criteria except ACR50 and ACR70 (although a trend for a poor response was observed) [25]. It has also been shown that smokers tend to consume a higher number of DMARDs over time, suggesting that smoking can reduce therapeutic efficacy and that non-smokers are more likely to achieve an ACR response than smokers [51]. As proposed by Saevarsdottir *et al*., smoking may interfere with the pharmacodynamic and pharmacokinetic properties of the drugs, thus altering responsiveness [50]. Stamp *et al*. showed that the intracellular levels of some MTX polyglutamates were decreased in smokers [52], suggesting that MTX metabolism is altered which leads to a poor response. Whatever the mechanism, active smoking is an important modifiable factor that seems to be associated with a poor response to MTX. Tobacco discontinuation should be encouraged and considered an important part of the therapeutic approach.

Longer disease duration has been identified by Anderson and colleagues as the most important factor to predict worse response to MTX in the extensive meta-analysis mentioned before [24], and similar findings were reported in other publications, regarding both MTX and other DMARDs [16,25,32,33,37,53,54]. However, Hoekstra *et al. *[23] failed to demonstrate such an association in a RCT comprising 411 patients treated with MTX (although the mean disease duration was lower) and several other studies also did not detect that association with MTX and other DMARDs [14,15,22,27,28,55-57]. It has been widely shown that treatment of early RA yields better results than treatment of established disease [6,58-61] leading to the 'window of opportunity' concept [62-64]. Discrepancies in these results might have been induced by evaluations performed mostly in established RA patients, who probably have a more uniform response to MTX, or in early RA populations that have short-term disease and a narrow disease-duration span making it harder to detect differences in response rates. Thus, while it is likely that patients with early disease respond better than those with established RA, disease duration seems to lose its negative influence with long-term progression of disease and this might confound the results of studies addressing this factor.

An additional question is whether the worse response to treatment in established RA patients is a direct consequence of longstanding disease by itself or if it is related to the failure of previous DMARDs, as was previously discussed by Hider *et al*. in their 2005 review [16]. Despite the existence of a few reports suggesting that previous DMARD use does not affect response to further treatments [28,55,57], most evidence seems to point in the opposite direction. In fact, literature findings include references to a negative effect of previous DMARD use on the response to treatment with MTX and other DMARDs [24], shorter time to DMARD discontinuation in patients who had previously taken MTX [53] and lower drug survival for DMARDs started following previous therapy and late in the course of the disease [65]. Likewise, Lie *et al*. found that patients who had previously taken other DMARDs had significantly lower response rates to MTX monotherapy [66]. Based on this study, the absence of any past DMARD therapy was identified as one of the predictive factors of a favorable response to MTX monotherapy [15]. Similar findings were reported by Aletaha and colleagues in patients taking consecutive DMARD courses, with the first DMARDs obtaining a greater decrease in C reactive protein (CRP) than subsequent ones [1]. Another study found that the effectiveness of a particular DMARD was always higher when started after non-steroidal anti-inflammatory drugs (NSAIDs) than after another DMARD [67]. It may be postulated that patients who do not respond to a certain drug might have a globally more severe and less responsive disease, but other mechanisms might explain these observations. As proposed by Hider *et al. *[16] previous therapies may alter drug kinetics and influence metabolism in such a way that the effectiveness of subsequent drugs can be lowered. However, this hypothesis has not been adequately tested so far.

Disease activity at baseline has been thoroughly investigated as a potential marker of response but globally the results are inconsistent, which can be related to the different clinical instruments and response criteria used in the studies. In fact, disease activity can be assessed by isolated clinical-laboratory variables (CRP, erythrocyte sedimentation rate (ESR)), tender joint count (TJC), swollen joint count (SJC), global assessment of disease activity on a visual analogue scale (VAS) or by composite scores (DAS, DAS28, CDAI, simplified disease activity index (SDAI)) and different criteria are used to define response (EULAR, ACR, DAS/SDAI remission). Thus, it is crucial to consider this information when interpreting literature data. In the meta-analysis by Drouin *et al*., high disease activity at baseline as measured by DAS or SDAI was identified as a predictor of a weak response to MTX monotherapy [15]. Wessels *et al*. showed that in an early RA population, high DAS and high SJC were associated with a poor response to MTX monotherapy, defined as achieving a DAS ≤2.4 at 6 months (OR = 0.1, 95% CI 0.0 to 0.1) [22]. Other factors such as VAS, ESR and CRP did not seem to have an effect on response. In an established RA study, higher disease activity defined by DAS was also related to a decreased likelihood of response to MTX (OR = 0.53, *P *<0.001) [23]. These results are in accordance with the findings of Aletaha *et al*., that early RA patients with higher baseline SDAI (but also CDAI and DAS28) were less likely to achieve remission or low disease activity at one year of MTX monotherapy [68]. In this study, it was observed that the association between disease activity and remission at one year was low but significant at baseline and increased greatly in subsequent visits, with SDAI at three months being highly predictive of one-year remission. Similarly, Saevarsdottir and colleagues found that higher DAS28 at the moment of MTX start predicted a lower likelihood of EULAR response (OR = 0.64, 95% CI 0.52 to 0.80), despite no significant association being shown for ACR, SDAI or CDAI response criteria [25]. Vázquez *et al*. demonstrated that in early RA, patients with low to moderate disease activity at baseline (DAS28 <5.1) were four times more likely to be in remission (DAS28 <2.6) after two years of MTX ± gold therapy. Two other studies also demonstrated that in patients with recent onset RA treated with MTX, SSZ or both, a lower baseline DAS was predictive of remission at two [69], three and five years [70]. Thus, the literature seems to show that when disease activity is assessed by composite measures, lower activity at baseline predicts better responses to MTX. Despite this, baseline DAS28 was not different between responders and non-responders to MTX monotherapy in other early [14] and established [28,71] RA studies.

When disease activity is determined by isolated laboratory and clinical variables, evidence is much weaker and somewhat inconsistent. Anderson *et al*. found lower patient, but not physician, global assessment at baseline to be predictive of worse response to MTX and other DMARDs, a result that contradicts the data presented above. Most studies, however, did not find isolated patient/physician global disease assessment to influence response to treatment with MTX [22,27,29,66,71] or other DMARDs [27,29,34,45]. Wessels *et al*. showed high SJC to predict poor response to MTX in early RA [22], a finding not confirmed in established RA [66]. Ma *et al*. determined that a TJC higher than 5 at baseline decreased the likelihood of achieving DAS remission at 24 months, with no effect observed for SJC [29]. Verstappen *et al*. identified a lower Thompson joint score [72] at baseline as predictive of remission at 62 months in patients treated with MTX, gold or HCQ [73]. However, SJC and TJC as isolated variables were also shown not to be predictors of response to treatment with MTX and other DMARDs in several studies [14,24,27,28,34,70,71]. As a whole, these data suggest that low disease activity defined by isolated clinical variables is probably associated with a better response to treatment, which is in accordance with the above results for composite measures. However, they should not be used as independent response predictors because comprehensive scores, such as DAS or SDAI, are better predictive tools. Similarly, inflammatory markers are sometimes used to assess disease activity but, globally, results are also far from being in consensus. In the meta-analysis by Drouin [15], neither CRP nor ESR were predictors of response to MTX monotherapy. These conclusions were based on two studies, one of them identifying high ESR to be associated with a worse response in established RA [66] but the other, regarding early RA, only finding this association in the univariate analysis [22]; both showed no effect of CRP. Other studies regarding therapy with MTX ± other DMARDs did not show any effect of ESR and/or CRP on response to treatment [14,24,26-29,70,71]. On the other hand, the study by Combe *et al*. identified ESR and CRP as two of the five independent predictive factors of disability at five years in early RA patients treated mainly with MTX and SSZ [42]. As to other DMARDs, Matteson *et al*. found that ESR did not influence response to HCQ monotherapy [34] but in another study a low baseline CRP was the only predictor of a favorable response to HCQ monotherapy in early RA patients (OR (CRP ≤10 mg/L) = 3.6, 95% CI 2.2 to 6.0) [35]. van Roon and colleagues identified ESR <35 mm.h^-1 ^at treatment start to predict higher leflunomide survival (hazard ratio (HR) = 1.38, 95% CI 1.01 to 1.88) [36] and likewise, high ESR at disease onset and at DMARD initiation predicted early discontinuation of treatment in an established RA study (HR = 1.05 per 10 mm.h^-1 ^increase, 95% CI 1.02 to 1.08) [53]. Contrary to these findings, Capell *et al*. observed that a lower ESR was related to a worse response to gold, penicillamine or SSZ [32]. As a whole, these results are not sufficient to state whether ESR or CRP alone are predictive factors of response to MTX and other DMARDs. While some studies showed a significant association between inflammatory markers and response, usually with higher baseline values associated with weaker treatment responses, others, including large meta-analyses, do not find these variables to be good predictive markers, at least when considered independently. In the light of current evidence, for the purpose of predicting DMARD response, it is probably better to integrate ESR and CRP components as part of disease activity scores and not judge them individually.

Disease severity and disability at baseline were also proposed by some authors as being predictive of treatment response. Anderson *et al*. identified a lower functional status, according to the Steinbrocker criteria, to be associated with a weak response to MTX and other DMARDs [24]. In two early RA studies, patients treated with MTX, SSZ or both were more likely to be in remission (DAS <1.6) at two [69] or three years [70], if they had a low baseline health assessment questionnaire (HAQ) score. Similarly, in other early RA studies, a high HAQ at baseline predicted a poorer response to MTX monotherapy [22,25,45] and to a combination with HCQ [45], with HAQ being the only significant predictor of remission, using all remission definitions, in the paper by Saevarsdottir *et al*. (OR = 0.56, 95% CI 0.40 to 0.80) [25]. However, several studies showed contradictory results, with baseline HAQ not being an independent predictor of responsiveness to MTX [14,27-29,66,71,74] and other DMARDs [27,28,34,35,74]. While some studies seem to suggest that a higher HAQ predicts a weaker response to MTX and other DMARDs, several other studies with similar populations did not confirm this association.

Pain score was not identified as a predictor of clinical response to MTX monotherapy in the review by Drouin *et al. *[15] but, contrarily, Goetz and colleagues concluded that a higher baseline pain score was associated with poor response to therapy [75]. In fact, while some studies identified high pain scores to be associated with lower four-year remission rates after treatment with MTX, gold or HCQ [73] and lower responsiveness to HCQ [34], most authors were not able to define baseline pain as an independent predictor of clinical response to MTX [22,24,27,29,45,66,69-71] and other DMARDs [24,27,35,69,70], either in early or established RA. Thus, it seems that evidence suggests that baseline pain scores are not independent predictors of response to DMARD treatment.

Other factors have been studied and there are scattered reports proposing them as possible predictors of response. Concomitant NSAIDs use was associated with an increased efficacy of MTX monotherapy in established RA [23] and a similar significant but weak association was seen in early RA (OR = 1.31, 95% CI 0.84 to 2.06) [25]. Most studies did not analyze the effect of NSAIDs and so, given the small amount of evidence, further studies are needed to confirm this association, although a beneficial effect may be expected. Results on concomitant corticosteroid therapy are more difficult to interpret because of different doses and timings for starting steroids (before DMARD therapy, during, or both). Saevarsdottir and colleagues found that early RA patients who were already on stable low-dose prednisolone at the start of MTX responded better (OR = 2.84, 95% CI 1.43 to 5.63) [25] and Hider *et al*. showed that absence of steroid use predicted MTX inefficacy at two years, but not at one year [14]. These results are in accordance with trials that showed that patients treated with combination therapies including steroids have better responses than those on DMARD monotherapy [76-79], even though in these studies steroids and DMARDs were started simultaneously. However, other studies found no association between corticosteroid use and DMARD response [24,34]. Despite these latter observations it seems likely that patients on corticosteroid concomitant treatment are more likely to respond to DMARD therapy. Erosion and radiographic scores at baseline do not seem to be reliable predictors of treatment response to DMARDs as was shown in several reports [27,29,35,45,53,69,73,74].

## Nongenetic biomarkers of response

Among nongenetic biomarkers, autoantibodies are probably the most important and most studied (Table 3). In fact, RF and anti-citrullinated protein antibodies (ACPA) are important markers with diagnostic and prognostic roles in RA and were both included in the 2010 RA classification criteria [80]. RF is associated with persistent disease and radiographic progression [21,81-83] but its role in predicting response to treatment is less clear. A large number of studies, comprising a considerable number of patients, showed that RF status does not predict response to MTX and other DMARDs in both early and established RA [1,14,23,25,27-29,34,36,37,42,45,53,55,66,84]. However, in the study by Wessels *et al. *[22] RF-positivity alone presented a trend towards worse response to MTX monotherapy in early RA patients; RF-positive smokers were definitely worse responders. Similarly, in a retrospective study with 265 patients, Morgan and colleagues found that resistance to three or more DMARDs was more frequent in RF-positive patients (OR = 2.15, 95% CI 1.00 to 4.62) [85]. Verstappen *et al*. found RF-negativity to be associated with four-year remission in early RA patients started on HCQ, MTX or gold (β = 1.63; *P *= 0.061) [73]. Some authors found RF-positivity to be associated with lower remission rates [70,81,86-89]. However, these studies analyzed remission as an outcome and, thus, these results were more likely to be directly related to the role of RF as a marker of more persistent and severe disease, and not necessarily linked to treatment effectiveness. Overall, most of the available evidence seems to show that baseline RF status does not influence the effectiveness of DMARDs.

**Table 3:** Summary of nongenetic biomarkers of response to MTX and other DMARDs.

Factors	Predictors of response?k	Comments
RF	No	Some results confounded by its poor prognostic role; however, most evidence is clear in that it does not influence treatment response
ACPA	Not likely	More data needed but does not seem to predict response; associated with worse outcomes in some studies but may reflect more severe disease; interesting reports in UA, pending confirmation
Anti-MCV	Unknown	Suggested to relate to more severe disease; not yet addressed in terms of response to treatment
Creatinine clearance	No	Few studies analyzed this factor, no association with MTX response in a meta-analysis
Hb levels	Uncertain	Anecdotal reports of association with better response; needs confirmation and its role should be clarified in future studies
Cytokines	Uncertain	Small/pilot studies suggesting association with response; potential promising role of baseline TNF levels, TNFID_50 _and IL-1ra/IL-1β ratio
Others	Uncertain	Other interesting factors analyzed in small studies and not further confirmed include MMP-3, urinary 7-OH-MTX and IgG hypogalactosylation

Conclusions and comments are based on the findings reported and discussed in the text. ACPA, anti-citrullinated protein antibodies; anti-MCV, anti-modified citrullinated vimentin antibodies; DMARDs, disease modifying anti-rheumatic drugs; Hb, hemoglobin; IgG, immunoglobulin G; IL-1ra, interleukin-1 receptor antagonist; IL-1β, interleukin-1β; MMP-3, matrix metalloproteinase-3; MTX, methotrexate; RF, rheumatoid factor; TNF, tumor necrosis factor; TNFID_50_, dose required to suppress by 50% the production of tumor necrosis factor; UA, undifferentiated arthritis; 7-OH-MTX, 7-hydroxy-methotrexate.

The presence and levels of ACPA are currently very important in the diagnosis and prognosis of RA. Diagnostically, they are highly specific (higher than RF) and have a good sensitivity (equal or slightly lower than RF) [90-92]. In terms of prognosis they are associated with worse functional status [93,94], higher disease activity [95,96], severe radiographic progression [13,97-104] and worse disease course [104-106]. Data are much scarcer than with RF, but two early RA studies (n = 205 and n = 405, respectively) showed that ACPA did not influence MTX effectiveness [22,25]. The study by Cao *et al*. also found no differences in second-line DMARD response between ACPA-positive and ACPA-negative patients from an early RA cohort that had previously failed first-line DMARD therapy [107]. Likewise, Hodkinson *et al. *[45], Verschueren *et al. *[108], Vázquez *et al. *[27], Boire *et al. *[109], da Mota *et al. *[84] and Gossec *et al. *[70] found no association between ACPA status and the likelihood of achieving low disease activity or remission at 1, 2, 2.5, 3 or 5 years in DMARD-naïve early RA patients treated with MTX and/or other DMARDs. A recent subanalysis at 8-years follow-up of the BeSt study (n = 484) specifically addressed the association of ACPA with treatment response. The analysis of all treatment groups as a whole, including one arm starting with infliximab [110], showed that ACPA-positive patients responded as well as those who were ACPA-negative, with similar decreases in disease activity, remission rates and functional ability, although they had worse radiographic progression and were less likely to maintain drug-free remission. This last finding was reproduced by other authors who found ACPA positivity to be associated with inability to maintain drug-free remission for more than one [111] or five years [93]. However, there are some contradicting results. In a study comprising 124 Japanese patients treated with MTX or SSZ within one year of disease onset, ACPA positivity was strongly associated with resistance to treatment (OR = 6.31, *P *= 0.027), but the criteria used to define non-responders (starting anti-tumor necrosis factor (TNF) agents during two years of follow-up) was different from other studies and this must be taken into account [112]. Verstappen *et al. *[113] recently found the presence of ACPA to be strongly associated with initiation of biological therapy in an early inflammatory polyarthritis population, although this was not directly linked to failure of initial DMARD therapy and may represent the effect of a more severe disease that will require more aggressive therapy. On the other hand, there are some studies that identified an association of ACPA with decreased likelihood of achieving remission at two [87] or eight years [89] in recent onset RA. Other studies have also found a lower response to treatment in ACPA-positive patients, in terms of the magnitude of decrease in DAS28, ESR, CRP and other clinical variables [102,105,114]. This may be just a reflection of the higher disease activity that characterizes ACPA-positive disease and it is difficult to state with certainty that it represents a worse response to the treatment instituted. Another issue raised by van Dongen *et al. *[115] and already reported by others [19] concerns the beneficial effect of MTX in delaying the progression to RA in ACPA-positive undifferentiated arthritis (UA) but not in ACPA-negative UA. This would suggest a favorable effect of ACPA in terms of response to therapy, but the follow-up analysis showed that in ACPA-positive patients, non-responders had higher pretreatment ACPA levels, a finding confirmed in a similar population of patients from the BeSt study [116]. Although the number of patients was small, these results would suggest that while MTX is more effective in UA patients who are ACPA-positive and, thus, probably at a higher risk of developing RA, the titer of this antibody is inversely related to the response to MTX. Larger studies are needed to confirm this potential effect. Overall, evidence does not support the role of ACPA as predictive markers of response to MTX and other DMARDs. Despite some opposite results in terms of remission and response to treatment, the strong prognostic value of this marker associated with worse disease outcomes must be taken into account when analyzing the results and can sometimes disturb the distinction between poor response to treatment and poor prognosis *per se*. Interesting findings on UA need further confirmation with larger populations and, as suggested by Visser *et al. *[116], pretreatment ACPA levels should be obtained in studies analyzing response to treatment.

The evidence presented above for ACPA was based on studies using the most common assay, the second-generation anti-cyclic citrullinated peptide (anti-CCP2). Recently, another test targeting modified citrullinated vimentin (anti-MCV) was developed to identify a particular member of the ACPA family [117]. While its current role is not clearly defined in RA, it seems to be as good as anti-CCP2 as a diagnostic marker, with sensitivity and specificity of 62% to 84% and 83% to 95%, respectively, being reported in the literature [118-122]. However, in terms of prognosis there are contradicting results. Anti-MCV was associated with more severe and erosive disease in some studies [109,120,123,124], with a clear correlation with disease activity. In this case, high anti-MCV levels could relate to more active disease and possibly lower response to treatment. However, neither this association [119,122,125-128] nor this correlation was observed by other authors [126-128] and to our knowledge, the impact of anti-MCV status on response to DMARD treatment was not specifically addressed in the studies published to date. It would be of interest to further analyze the role of this antibody as a potential predictive marker of response in future investigations.

Other biomarkers have been studied as potential predictors of response to treatment. ESR and CRP have been discussed earlier, as markers of disease activity.

Creatinine clearance was inversely related to MTX efficacy in one established RA study [23] but the association was weak (OR = 0.99, 95% CI 0.98 to 1.0) and no statistically significant difference was observed in other studies [22], including a meta-analysis evaluating 11 RCT (n = 496) that specifically addressed the influence of renal function (and age) on MTX responsiveness [39]. Serum creatinine levels were also demonstrated to be non-predictors of leflunomide treatment survival [36] but data on other DMARDs are even scarcer. High hemoglobin levels were associated with remission (DAS28 <2.6) at two years in the univariate analysis in early RA, DMARD-naïve patients. However, it was not an independent predictor of remission when assessed by multivariate logistic regression analysis [27]. In a recent study with a similar population, high hemoglobin levels independently predicted a low disease activity state (SDAI <12) after two years of therapy with MTX ± SSZ ± chloroquine sulfate [45]. Although most studies regarding treatment response do not analyze the role of baseline hemoglobin levels as a potential predictive marker, it is known that persistent inflammation can lead to anemia, particularly through the action of IL-6 [129], and low hemoglobin levels have been associated with more active and severe RA [130]. Thus, while hemoglobin concentration may constitute an indirect marker of disease activity, caution is required when interpreting response to treatment, because the suggested association of high hemoglobin and a low disease activity at follow up may simply reflect a milder disease, with less inflammation, lower activity scores and not necessarily a true correlation with better DMARD effectiveness. Nevertheless, as a simple, cheap and widely available laboratory variable, it would be interesting if upcoming studies analyzing treatment response include hemoglobin levels and further investigate whether it may play a true role as a predictive marker.

Being involved in the pathogenesis of RA [131], cytokines are also influenced by MTX and other DMARDs [132,133]. Therefore, they are an appealing potential biomarker of response to treatment and have been evaluated in some studies. Baseline serum concentration of TNF was inversely associated with six-month response to MTX and other DMARDs, and levels below 20.1 pg/mL could independently predict responders with high specificity and sensitivity, in a small sample of both early and established RA patients (n = 38) [28]. No effect was seen regarding IL-1β, IL-6, IL-8, IL-10 and IL-12. However, in a study of 50 consecutive established RA patients who had already failed one to three DMARDs, pretreatment serum TNF and IL-1β levels were undetectable in the majority of patients and did not predict response to treatment; neither did serum levels of IL-1 receptor antagonist (IL-1ra) or soluble TNF receptor (sTNFR) p55 [134]. In this study, though, a significant association was seen between a low IL-1ra/IL-1β synthesis ratio of unstimulated pretreatment peripheral blood mononuclear cells (PBMC) and good/excellent responses to MTX: an IL-1Ra/IL-1β ratio lower than 100 strongly predicted an ACR response higher than ACR50 (*P *<0.0001), with positive and negative predictive values of 94% and 91%, respectively [134]. It has also been demonstrated that baseline IL-10 production by PBMC was higher in MTX responders than in non-responders [135]. Another study comprising a reduced number of early RA patients (n = 8) reported a correlation between a higher baseline percentage of IL-4 positive CD4+ T cells and low disease activity at six to nine months of MTX treatment [136], a finding in line with other data suggesting a relationship between a low IFN-/IL-4 ratio and better nine-month response to HCQ and SSZ [137]. Low pretreatment levels of soluble IL-2 receptor (sIL-2R, <442 U/mL), translating reduced T-cell activation, predicted six-month remission in early RA patients treated with SSZ monotherapy [138], an association not confirmed in patients with established disease treated with MTX (mean disease duration >10 years) [139] or SSZ/gold (mean disease duration >5 years) [140]. A small study reported that in 14 early RA patients treated with HCQ, soluble CD30 (sCD30) basal levels were higher in responders than in non-responders (*P *<0.03), which might be related to a higher activity of Th0/Th2 anti-inflammatory cells [141]. This was not confirmed in a study of 92 RA and UA patients treated with MTX and other DMARDs, where baseline sCD30 levels did not associate with treatment response at one year [142]. In a pilot experimental study (n = 25), the MTX-induced *in vitro *inhibition of T-cell cytokine production was studied and a strong negative correlation was found between clinical response at four months and the dose required to suppress by 50% the production of TNF (ID_50_, r = -0.62, *P *<0.01) [143]. Patients with a TNFID_50 _lower than 224 ng/mL had a significantly greater reduction in DAS28 after four months of MTX treatment than those with a value above this cutoff (*P *<0.02), which had a sensitivity of 93% and a specificity of 86% for predicting patients with a moderate EULAR response. Since the effect was so pronounced, *in vitro *suppression of TNF is an interesting assay that may predict response to MTX and guide individual therapeutic decisions; to date and to our knowledge, validation in a larger cohort is pending. As a whole, the data concerning cytokines confirm their potential as predictors of treatment response. Despite the elevated cost associated with most of these assays, they may enable individualized therapy in RA patients if clear associations are confirmed in other, larger, studies.

Reports concerning other biological markers are available but no definite conclusions can be taken regarding their true role as predictive markers, because most studies were pilot studies, based on small samples and their findings were not further validated. These include hypogalactosylation of IgG [144], serum matrix metalloproteinase-3 (MMP-3) levels [145,146], urinary levels of the less effective MTX catabolite, 7-hydroxy-MTX [147], red blood cell (RBC) levels of MTX polyglutamates (MTX PG), the active anti-inflammatory metabolites of MTX [148-152] and synovial vascularity [153].

## Genetic biomarkers of response

Pharmacogenetics may provide an objective explanation for the discrepancies observed in response to DMARDs among patients: the genetic characteristics of each patient might interact with a certain drug, interfere with its pharmacokinetics or target, thus affecting its pharmacological action and ultimately leading to different effects. Intense efforts have been focused on the pursuit of polymorphisms and genetic patterns that associate with increased or decreased drug response and the major findings are summarized in Table 4.

**Table 4:** Summary of genetic biomarkers of response to MTX and other DMARDs.

Factors	Predictors of response?	Comments
SE	Yes, worse response to MTX	SE-positive patients seem to respond worse to MTX, especially carriers of the HLA-DRB1*04 allele; association with remission controversial; not extendable to other DMARDs
*RFC1 *	Likely, better response to MTX	80G>A: evidence suggests favorable response in variant allele carriers, although some studies did not confirm it; other identified SNPs may have a role and explain discrepancies
*ABCB1 *	Uncertain	3435C>T: several studies suggesting association with better response, not confirmed in others
*ABCC1-4*/*ABCG2*	Unknown	Not thoroughly studied
*GGH*	Uncertain	Conflicting results regarding SNPs 401C>T, 452C>T and 16T>C
*FPGS*	Uncertain	Few studies; contradicting findings with SNP 14G>A, no association of 14G>A with response in two studies
*TYMS*	Uncertain	TSER *R/*R: opposite results suggesting better responses for both 2R/2R and 3R/3R and others showing no effect
		6 bp-del: favorable role suggested, but not found in other studies
*DHFR*	Uncertain	Several SNPs described but addressed in single studies; 317A>G was the only one associated with response but only when using rDAS28 and with marginal effect
*ATIC*	Uncertain	347C>G is the most studied, but conflicting results did not allow a definition of its role; other SNPs have been identified and associated with response in a few studies but lack replication
*MTHFR*	No	677C>T and 1298A>C have been extensively studied and two large meta-analysis found no association with MTX effectiveness
Polygenic combinations	Uncertain, but promising	Several reports of SNPs combinations associated with response, but lacking replication

Conclusions and comments are based on the findings reported and discussed in the text. ABC, ATP-binding cassette (B1, C1-4 and G2); ATIC, 5-aminoimidazole-4-carbox-amide ribonucleotide transformylase; DMARDs, disease modifying anti-rheumatic drugs; DHFR, dihydrofolate reductase; FPGS, folylpolyglutamate synthetase; GGH, **γ**-glutamyl hydrolase; HCQ, hydroxychloroquine; HLA, human leukocyte antigen; MTHFR, 5,10-methylene-tetrahydrofolate reductase; MTX, methotrexate; rDAS28, relative disease activity score - 28 joint; RFC1, reduced folate carrier 1; SE, shared epitope; SNPs, single nucleotide polymorphisms; TYMS, thymidylate synthase.

*HLA-DRB1 *shared epitope (SE) alleles are well-established risk factors for RA [154,155] and are associated with more severe and erosive disease [156-159]. However, their influence on DMARD effectiveness is not clear despite several studies that have tried to approach this question. O'Dell *et al*. showed that SE-positive patients, who had previously failed one DMARD, were much more likely to obtain ACR50 responses if they were on combination treatment (MTX plus SSZ plus HCQ) compared to MTX monotherapy (94% and 32% responders, respectively; *P *<0.01), with no difference being seen in patients who were SE-negative (n = 84) [55]. Additionally, patients on MTX monotherapy responded better if they were SE-negative (83% and 32%, respectively, *P *<0.04), an effect that was lost in the combination treatment group. Similarly, Ferraccioli and colleagues demonstrated that, at six months, *HLA-DR 0401*-positive patients responded worse to MTX monotherapy (29% and 80% responders, respectively) and better to cyclosporine (52% and 5.8%, respectively) than those who were *HLA-DR 0401*-negative [160]. Hider *et al*. studied 309 patients from an inception cohort with inflammatory polyarthritis and found that the possession of the *HLA-DRB1 *SE was the only factor predicting MTX monotherapy inefficacy at one and two years, with a strong association (adjusted OR = 5.88 and 3.04, respectively, both *P *= 0.02). In the recent Japanese study mentioned above [112], early RA patients carrying one or two copies of the SE-positive HLA-DRB1*04 allele (especially *0405) were more likely to be resistant to DMARD therapy (predominantly MTX) at two years (OR = 2.89, *P *= 0.011), an effect not seen with other SE-positive alleles. In line with these findings, González-Gay *et al*. previously reported that patients positive for SE alleles were significantly more likely to be treated with cyclosporine A, because of insufficient response to MTX or MTX plus chloroquine (OR = 2.9, *P *= 0.006); the strongest risk for requiring cyclosporine A treatment was seen with the HLA-DRB1*0401/*0404 genotype [161]. Yet, in a Pakistani population of 91 RA patients, the only SE allele associated with response to treatment was HLA-DRB1*03, significantly more common in non-responders, with no effect being observed in other alleles, including DRB1*04 and DRB1*01 [162]. As a whole, these studies indicate that SE-positive patients may respond worse to MTX and that the HLA-DRB1*04 allele (and maybe also HLA-DRB1*03) plays an important role in this effect. However, several authors failed to show an association between SE-status and induction [27,37,70] or persistence [163] of remission in patients treated with MTX and other DMARDs, while others found the absence of SE alleles to be associated with DMARD-free remission [111] or remission to be more likely in patients with ≤1 SE-allele [86]. Few studies have analyzed other DMARDs in monotherapy regimens [34,164]. Globally, SE seems to influence response to DMARD treatment, with an apparent negative effect on MTX response, and further studies analyzing predictors of response should include this genetic marker in order to clarify its true influence on drug effectiveness.

**Figure 1:**
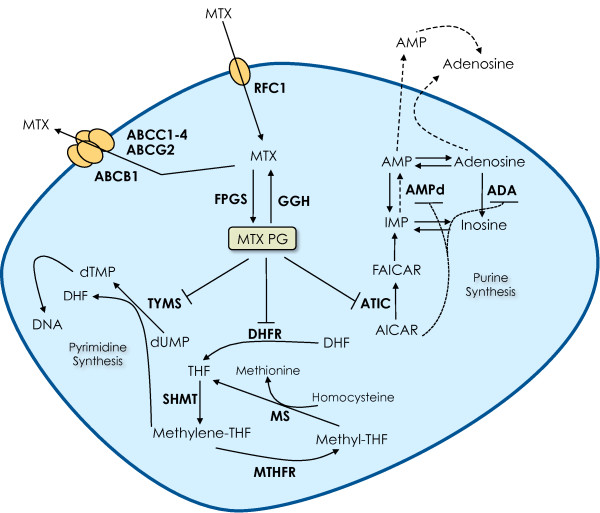
**Methotrexate mode of action**. Methotrexate (MTX) is actively transported into the cell by the reduced folate carrier 1 (RFC1; also known as SLC19A1) and is then polyglutamated by folylpolyglutamate synthetase (FPGS) to form MTX polyglutamates (MTX PG), which are kept inside the cell [221] and are responsible for MTX anti-inflammatory intracellular actions [17,174]. Glutamates can be removed by **γ**-glutamyl hydrolase (GGH) and MTX monoglutamate is rapidly effluxed from the cell via membrane transporters of the ATP-binding cassette (ABC) family [222], especially ABCC1-4 and ABCG2 [223,224]. Inside the cell, MTX PG exert their anti-inflammatory actions through inhibition of essential enzymes of the folate pathway: dihydrofolate reductase (DHFR) [225], blocking the conversion of dihydrofolate (DHF) to tetrahydrofolate (THF) and ultimately leading to depletion of methionine and decreased DNA methylation; thymidylate synthase (TYMS) [226,227], interfering with *de novo *pyrimidine synthesis; and 5-aminoimidazole-4-carbox-amide ribonucleotide (AICAR) transformylase (ATIC) [148,228], an enzyme of the *de novo *purine synthesis, causing accumulation of AICAR, which will finally result in increased secretion of adenosine, a strong anti-inflammatory mediator [229,230]. The enzyme 5,10-methylene-tetrahydrofolate reductase (MTHFR) is not directly inhibited by MTX, but is affected by it because of its action in the folate pathway [176]. ADA, adenosine deaminase; AMPd, adenosine monophosphate deaminase; dTMP, deoxythymidine monophosphate; dUMP, deoxyuridine monophosphate; FAICAR, 10-formyl 5-aminoimidazole-4-carboxamide ribonucleotide; IMP, inosine monophosphate; Methyl-THF, 5-methyl-tetrahydrofolate; Methylene-THF, 5,10-methylene-tetrahydrofolate; MS, methionine synthase; SHMT, serine hydroxymethil transferase.

Figure 1 illustrates the MTX cellular pathway and mode of action. Single nucleotide polymorphisms (SNPs) in genes codifying proteins involved in this process have been identified and thoroughly studied for their influence on the response to MTX.

Regarding membrane transporters, *reduced folate carrier 1 (RFC1*) 80G>A may influence influx of MTX into the cell, but its influence on drug responsiveness is not clear. It has been reported that patients with the *RFC1 *80A/A genotype have a greater response to MTX (based on several disease activity measurements) than wild-type 80G/G patients: lower global VAS [165], lower SJC and disease activity VAS [149], better EULAR responses [166] and a 3.32-fold higher probability of achieving remission (*P *= 0.021, n = 174), with statistically significant differences in the A allele prevalence between good and poor responders (62.1% and 47.8%, respectively, *P *= 0.013) [167]. Other relevant findings include higher RBC MTX PG levels in AA homozygous RA patients compared to other genotypes (*P *= 0.007) [168], higher MTX plasma levels in AA children with acute lymphoblastic leukemia (*P *= 0.004) [169] and lower uptake of MTX in CD4+ T cells and B cells in healthy individuals expressing the GG genotype, compared to those having the A allele [170]. These arguments seem to support a favorable role for the *RFC1 *80G>A SNP as a predictor of good response to MTX, but other authors have failed to confirm its association with MTX efficacy [150,152,171-173]. Caution must be taken regarding interpretation of RBC MTX PG levels, because currently it is not absolutely certain that they represent the actual concentration of these metabolites inside other important cells in RA such as leucocytes or synovial cells [174]. Furthermore, a recent study identified six other SNPs in the *RFC1 *gene associated with poor response to MTX, which contributes to the hypothesis that other polymorphisms in this gene may also affect the response to MTX, thus providing an explanation for the contradictory results in some of the studies [173].

*ATP-binding cassette (ABC) B1 *(*ABCB1*) codifies P-glycoprotein, a cell membrane-associated transporter of several drugs. Its influence on MTX efflux is unclear [175,176]. Nevertheless, the effect of *ABCB1 *SNPs has been studied, particularly that of 3435C>T. Pawlik *et al*. showed that patients with the 3435TT genotype were 2.89 times more likely to respond to MTX than those carrying one or two C alleles (n = 92) [177] and, likewise, Drozdzik and colleagues found a 4.65-fold higher probability of symptom remission in TT-positive patients, compared to those with the CC genotype (n = 174, *P *= 0.003) [178]. A recent study also reported lower mean DAS28 in 3435TT patients compared to the 3435CC genotype (*P *= 0.02) [152]. On the other hand, an Asian study found that patients with the 3435TT SNP were nonresponders more frequently than patients with the 3435CC genotype (adjusted OR = 8.78, *P *= 0.038) [172]. However, these differences may be related to the response criteria applied, which in this study were based on MTX maintenance dose (responders if dose was below 6 mg/week, nonresponders if MTX dose was above 6 mg/week) and not directly on the evolution of symptoms or disease activity. Additionally, other authors found no association between *ABCB1 *3435C>T and MTX efficacy [179,180]. Despite the description of frequent SNPs in other transport proteins involved in the efflux of MTX from the cell, such as ABCC1-4 and ABCG2 [181], they have not been thoroughly addressed regarding MTX effectiveness in RA; there are some reports of better response to MTX in psoriasis patients carrying SNPs for *ABCC1 *and *ABCG2 *[182] but recently three SNPs in *ABCC2 *and *ABCG2 *genes were not related to MTX response [152] and further studies are needed to clarify their true influence.

As to the enzymes involved in the glutamation cycle of MTX inside the cell, **γ**-glutamyl hydrolase (GGH) is the most studied one. The SNP 401C>T of the promoter region of *GGH *was shown to influence MTX PG levels, which were lower in patients carrying the TT genotype compared to those with one or two C alleles (OR = 4.8, *P *= 0.002) [168]. However, the same group of researchers did not find any effect of this SNP on response rates to MTX in a prospective longitudinal study (n = 48) [150]. Other SNPs in *GGH *include 452C>T, that has been associated with decreased enzyme activity and accumulation of intracellular MTX PG [183], but was found not to influence MTX efficacy [152,173,184]. The role of *folylpolyglutamate synthetase *(*FPGS*) has been less studied. Although its expression in PBMC has been associated with poor response to MTX [26], an unexpected result given the role it is thought to have in converting MTX to its active form, the SNPs of the *FPGS *gene identified so far are of unclear functional and clinical significance [176]. Two studies failed to demonstrate an association between the SNPs 14G>A and 1994A>G and MTX response [173,184], but Sharma *et al*. showed that carriers of the A allele of the 14G>A SNP responded worse (OR = 3.47, 95% CI 1.19 to 10.12) [185]. Thus, more data is needed to evaluate its influence on drug responsiveness.

MTX PG inhibit several enzymes, including thymidylate synthase (TYMS), dihydrofolate reductase (DHFR), 5-aminoimidazole-4-carbox-amide ribonucleotide transformylase (ATIC) and, indirectly, 5,10-methylene-tetrahydrofolate reductase (MTHFR) (Figure 1). TYMS is involved in the *de novo *synthesis of thymidylate, required for cell proliferation. A polymorphic tandem repeat sequence in the promoter region of the *TYMS *gene has been described, with a variable number of 28 bp repeats (TSER *R/*R) [186]. Dervieux *et al*. found *TSER *2R/2R patients responded better to MTX than patients with other genotypes, based on physician VAS [187]. Similarly, a Japanese study reported that patients with the *TSER *3R/3R genotype required higher MTX doses than those carrying at least one allele with two repetitions (*P *= 0.033) [188]. Another study also found that the 3R/3R genotype was associated with worse response to treatment, as part of a pharmacogenetic index comprising SNPs of other genes (*RFC1 *and *ATIC*) [149]. However, the same group found that after adjustment for MTX dose and RBC MTX PG levels, 2R/2R patients were less likely to respond, with no association being observed in unadjusted data [150]. Furthermore, James *et al*. reported better 12-month EULAR responses to MTX in patients carrying one or two 3R alleles treated with MTX, SSZ and HCQ [166]; other authors found no association between MTX response and *TSER *status [22,173,189]. Another polymorphism has been identified which consists of a 6-bp deletion at the 3'-UTR region of the *TYMS *gene [190] and is associated with decreased mRNA stability and expression [190,191]. Japanese patients homozygous for this deletion have been shown to have greater reductions in CRP (*P *= 0.0383) [188] and a lower MTX dosage required for a 50% decrease in CRP (β = -0.268, *P *= 0.039) [189] compared to other genotypes, although other studies did not find an association between this polymorphism and MTX response [172,173]. James *et al*. reported that Australian patients homozygous for the 6-bp deletion were all classified as responders (10 versus 0) and a similar non-statistically significant trend was seen for the 6-bp deletion allele as a whole [166]. These authors also found the 3R-del6 haplotype to be clearly associated with a better clinical response to MTX plus SSZ and HCQ (OR = 2.9, 95% CI 1.0 to 9.2) and defended the concept that while currently it is not known which polymorphism of the *TYMS *gene is better in assessing MTX efficacy, haplotype analysis should be used in future studies analyzing response to treatment [166].

DHFR is a major direct target of MTX PG and, as such, polymorphisms affecting its expression, function, or binding to MTX may interfere with response to treatment [192]. A Japanese study identified a SNP in the 3'-UTR of the *DHFR *gene (829C>T), with homozygous 829TT patients having significantly higher expression of the enzyme (*P *<0.001) [193]. However, the effect of this SNP on response to treatment is unknown and one study of a European population found patients to be 100% CC wild-type and thus no 829TT patients were detected [180]. Wessels *et al*. found no association between SNPs 473G>A or 35289G>A and MTX efficacy [171] as was the case in the study from James *et al*. on the 19 bp deletion in intron 1 of *DHFR *gene [166]. A recently published study analyzed the role of SNPs 216T>C and 317A>G and reported no difference in these genotypes between responders and non-responders, using the EULAR response criteria; the authors did find a significant difference, though, when using relative DAS28 (rDAS28, improvement related to baseline value) as a measure of MTX efficacy, with the 317AA genotype being associated with worse response (*P *= 0.05) [194]. Additional, larger studies are needed to confirm this association.

ATIC is the third enzyme directly inhibited by MTX and it is involved in the *de novo *purine synthesis and adenosine cycle (Figure 1). The most studied SNP has been the 347C>G and, as with other markers, results have been contradictory. Dervieux *et al*. identified the GG genotype to be associated with lower physician VAS, SJC [149,187] and TJC, as part of a pharmacogenetic index [149]; similar findings were found by Lee *et al*. using a proxy SNP in linkage disequilibrium with 347C>G (OR = 3.89, *P *= 0.01) [195]. However, these were cross-sectional studies analyzing disease activity at a single visit in patients treated with MTX and did not consider baseline scores. This might explain the differences with the findings reported by Wessels *et al*. in two analyses of the BeSt study population, in which patients with the wild-type homozygous 347CC allele were more likely to respond to MTX, after adjustment for baseline DAS28 [22,196]. Additionally, several authors found no association between 347C>G SNP and response to MTX [150,166,172,173,197]. Although differences might be related to different populations, stages of disease and study designs, it is currently unclear which is the true effect of this SNP on the effectiveness of MTX. Other SNPs have recently been identified with different associations to drug response [173,194]. Overall, *ATIC *polymorphisms seem to play a significant role in determining MTX effectiveness, which strengthens the great importance that adenosine is thought to have on the MTX mode of action.

Among all genes potentially influencing MTX efficacy, *MTHFR *is the best studied. This enzyme is responsible for the conversion of 5,10-methylene-tetrahydrofolate to 5-methyl-tetrahydrofolate, which is essential to the conversion of homocysteine to methionine [198]. Albeit not a direct target, it is influenced by MTX because of its effects on the intracellular folate pool [176]. Two SNPs have been thoroughly investigated in the last decade, with conflicting results. The 677C>T SNP leads to a thermolabile form of MTHFR with reduced activity [199] and among Caucasians around 50% carry at least one T allele [200], up to 40% are CT-heterozygous (60% enzyme activity) and approximately 10% are TT-homozygous (30% enzyme activity) [176]. The 1298A>C SNP also leads to reduced MTHFR activity, although less severely than the previously mentioned SNP [201], with the variant allele being present in 32% of the Caucasian population [202]. These polymorphisms seem to interact, as individuals heterozygous for both 677C>T and 1298A>C have greater decreases in enzyme activity, comparable to those homozygous for the 677C>T SNP [203]. Regarding the 677C>T SNP, although some authors reported better or worse six-month responses in patients with CC [171,204] or TT [150] genotypes, respectively, and higher rates of remission in carriers of the T allele [205], a striking number of studies showed no association between 677C>T and MTX efficacy [152,166,188,189,195,206-215]. In a similar way, data for the 1298A>C SNP is also controversial: while some authors found better responses in 1298AA-positive patients compared to other genotypes [152,171,204], others, on the contrary, reported that C-allele carriers had lower MTX maintenance doses [208,209] and a non-significant tendency for higher remission rates [205]. Most studies failed to identify an association between this SNP and response to MTX [150,166,188,189,210-215]. Moreover, to overcome the discrepancies observed for these polymorphisms two meta-analyses have recently been performed, which included a large number of studies and patients (n = 2,614 and 1,514, respectively), and in both the authors concluded there was no association between 677C>T/1298A>C and treatment response to MTX [213,216]. Thus, currently it is not possible to use *MTHFR *SNPs as reliable predictors of response to treatment.

Overall, studies evaluating the role of individual SNPs on response to MTX have been inconsistent. This may be related to different study designs, insufficient statistical power and several clinical and pharmacological confounders, such as ethnicity, outcome measures used, folate supplementation, MTX dose, duration and route of administration and concurrent therapies [174]. While large prospective studies are missing, meta-analysis may overcome this problem, but because there are numerous pathways and a considerable number of targets that can be affected by MTX, an individual genetic variant within a single gene is unlikely to result in a significantly altered response, enough to be detected and replicated in different studies. As such, it is probably more advantageous to address more than one gene and polymorphism simultaneously through polygenic analyses, haplotype analyses or gene-gene interactions. Other approaches include interaction of genetic and nongenetic factors and even, as proposed by Stamp *et al*., genome-wide association studies, which would obviate selection biases and might identify other potential predictors of response not included in current studies [174]. Examples of polygenic analysis include the studies by Dervieux *et al*., analyzing SNPs of different genes (*ATIC *347C>G, *TSER *2R/3R, *RFC1 *80G>A) as part of a pharmacogenetic index, a sum of the homozygous variant genotypes [149,187]. Higher indexes (that is, more variant SNPs) were associated with lower SJC, TJC and disease activity VAS [149,187], as well as with an increased probability of good response, with patients with at least one homozygous variant being 3.7 times more likely to have a good response to MTX than those with none (OR = 3.7, *P *= 0.01) [187]. Comparably, Wessels and colleagues investigated polymorphisms in genes involved in the adenosine pathway, such as *adenosine monophosphate deaminase *(*AMPD1*) 34C>T, *ATIC *347C>G and *inosine triphosphate pyrophosphatase *(*ITPA*) 94C>A, and found that patients carrying the *AMPD1 *34T allele, *ATIC *347CC or *ITPA *94CC had a greater likelihood of having a good response, which was significantly increased if all three favorable genotypes were present (OR = 27.8, 95% CI 3.2 to 250.0) [196]. These SNPs, alongside *methylene-tetrahydrofolate dehydrogenase (MTHFD1*) 1958G>A, were further included in a clinical pharmacogenetic model that proved to be effective in predicting response to MTX [22]. Haplotype analyses have been conducted in some studies. Urano *et al*. found the *MTHFR *677C-1298C haplotype to be associated with lower MTX dose (RR = 2.14, *P *<0.05), while no effect was seen in single locus analysis of the 677C>T SNP [209]. van der Straaten and colleagues found no association between individual SNPs and MTX efficacy, but identified patients with the *GGH *16C-allele and one or no copies of the *GGH *452C-16T haplotype as having good clinical improvement at three months, although they concluded that globally the SNPs tested were not likely to be predictive of treatment response [184]. Similarly, as mentioned above, James *et al*. found the 3R-del6 haplotype of the *TYMS *gene to significantly correlate with response to treatment [166]. These authors also demonstrated interaction between different SNPs, with patients having the *5-methyltetrahydrofolate-homocysteine methyltransferase *(*MTR*, which codifies for methionine synthase) 2756A allele in combination with either the *RFC1 *80A allele or the T*YMS *3R-del6 haplotype being 35 times more likely to respond to MTX plus SSZ and HCQ (*P *<0.0001) and 3.4 times more likely to achieve remission (*P *= 0.04) [166]. Hayashi *et al*. found patients with the *RFC1 *80AA genotype to respond better if they had no *GGH *401T alleles, suggesting an interaction between these two SNPs [215]. In the study by Sharma and colleagues a modest interaction, associated with MTX efficacy, was seen between *ABCB1 *3435C>T and *GGH *16T>C (*P *= 0.05) [217]. Dervieux *et al*. recently published two studies addressing this issue and found high-order interactions among SNPs in *RFC1*, *ITPA *and *ATIC *genes, which were associated with efficacy (3.89-fold lower likelihood of response in the absence of favorable combinations, *P *<0.001) [218]; on the other study, the authors reported an association among three interacting SNPs (*RFC1 *80G>A, *ITPA *94C>A and *ATIC *347C>G) and MTX efficacy (OR = 2.9, *P *<0.01), although it was not replicated in a different cohort [219]. While more studies are needed to reproduce these findings, as a whole, the analysis of different SNPs in various genes involved in the response to MTX and the interactions between them seems to be a promising approach that may bring more consistency to the body of data on MTX pharmacogenetics.

## Discussion

We found a high discrepancy between studies' results, making it difficult to obtain clear-cut predictors of response to MTX and other synthetic DMARDs (Tables 2, 3 and 4). This might be related to the heterogeneity in study types, population size and ethnicity, disease characteristics and outcome measures applied. While some factors (female gender, established disease, previous DMARD use, smoking, high disease activity determined by composite scores, absence of concomitant corticosteroids, SE-positivity) seem to be individually associated with a weaker response to MTX, drug effectiveness is ultimately the result of multiple clinical and biological (genetic and nongenetic) variables that interact to determine whether a patient responds or not to a particular drug. This explains why in most studies baseline factors merely associate weakly with better or worse response but individually fail to distinguish responders and nonresponders [15].

In line with this hypothesis, recent studies addressing different types of factors and the interaction between them have been shown to constitute a very promising approach to define, at drug start, which patients will respond and which will not [22,173]. In the study by Wessels *et al*., the authors applied a clinical pharmacogenetic score including clinical, genetic and nongenetic variables found to be associated with treatment response (gender, RF, DAS, smoking status, *AMPD1 *34C>T, *ITPA *94C>A, *ATIC *347C>G and *MTHFD1 *1958G>A) and correctly classified around 54% of the patients as responders or nonresponders (42% for responders and 63% for nonresponders); furthermore, scores of ≤3.5 had a positive predictive value of 95% and scores ≥6 had a negative predictive value of 86% [22]. When the genetic variables were removed from the model, the set of clinical and biological nongenetic factors correctly predicted response in only 29% of the cases (36% for responders and 23% for nonresponders), although positive and negative predictive values were still very high (89% and 92%, respectively) [22]. These findings clearly reinforce the notion that considering groups of potential predictive factors will be more efficient than simply analyzing them individually. Thus, including clinical, genetic and nongenetic biological factors is more effective than a parallel approach. Yet, this model has not been applied routinely in other populations or studies and to date few authors have replicated this type of approach, with most studies still focusing on searching for associations between individual markers and treatment outcome.

In this review, we were able to identify factors that seem to be associated with response to treatment, especially regarding clinical markers where the amount of evidence is greater (Table 2). Female gender, smoking, established disease, previous DMARD use, high disease activity measured by composite scores and the absence of concomitant corticosteroids are associated with a lower response to MTX. This is consistent with the results of the meta-analysis by Drouin *et al. *[15], with the difference that smoking was considered together with RF-positivity for early RA only and that corticosteroids were not shown to be predictive of clinical response to MTX.

A few comments should be made on these results. First, analyzing a significant number of studies for each factor may help overcome heterogeneity by giving a global view of the data and determining the direction evidence is pointing at; second, caution is required when interpreting results of individual negative studies; third, several studies consider MTX in association with other DMARDs, making it difficult to ascertain whether the observed effect in those cases is the result of MTX itself, the associated DMARD or the combination of both; and finally, given the paucity of studies specifically analyzing other DMARDs, it seems inappropriate to extend these conclusions to drugs other than MTX.

Biological markers, both genetic and nongenetic, have also been extensively studied. Despite some exceptions, most of the results lack confirmation and replication in larger studies (Table 3 and Table 4). The exceptions are RF, ACPA and SE, all thoroughly analyzed, given their role as predictors of poor prognosis. While most evidence points towards an absence of effect of RF on DMARD effectiveness (except for some early RA studies), SE-positivity (and especially the HLA-DRB1*04 allele) seems to be associated with a worse response to MTX (non-extendable to persistent remission). The presence of ACPA has a less well-defined effect, with solid data suggesting it does not influence response to DMARDs in early RA, although it may play a role in UA patients' response to MTX. Other nongenetic biomarkers have been identified in smaller studies and some may deserve further clarification as potential predictive markers through larger studies.

Pharmacogenetics remains a promising field but to date, and regardless of intense research, no SNPs have been clearly identified as predictors of response to MTX [17]. This is probably related to the influence of several genes and polymorphisms on the determination of the final drug effectiveness; recent studies continue to identify new SNPs in crucial pathways, underlining the complexity of this area. Valid approaches to overcome this issue include analysis of haplotypes, multiple-gene models, and interactions of different genes with nongenetic factors.

As previously mentioned, combining different factors might be useful in determining whether a patient will respond to MTX. Although models such as the one developed by Wessels *et al. *[22] might be more reliable for achieving this purpose, we can assume that starting and maintaining treatment with MTX will probably be more effective in male, non-smoking, DMARD-naïve, SE-negative patients with early, mild disease and that corticosteroids should be added as adjuvants. Other variables, such as genetic determinants, will be valuable in increasing the accuracy of the prediction model, but at the moment it is not possible to define them with certainty.

Standardization of studies addressing predictors of response is needed. On the one hand, the inconsistencies in results may reflect differences in study-design, population size and features (ethnicity, age, socioeconomic context), disease characteristics (early/established, activity, disability), pharmacological variables (dose, previous or concomitant DMARD, corticosteroids, NSAIDs) and, most importantly, outcome definitions used. On the other hand, this heterogeneity makes it harder to compare studies and, generally, systematic literature reviews and meta-analysis are forced to exclude a great number of studies, with few being left to analyze. In this review we included a wide variety of studies and while this is certainly a limitation it also allowed us to gather more data and have a broader picture of current evidence. Of major importance is definition of response, a question already raised by other authors [15,16,220]. Response to treatment may be defined as adequate symptom and activity control and this can be assessed by isolated clinical variables (VAS, SJC, TJC, pain VAS) or composite scores (SDAI, CDAI, EULAR response), but it must be taken into account that this is not the same as disease progression slowdown or halt. However, given the direct relationship between higher disease activity and progression, assessing response to treatment as improvement in activity or symptoms seems an adequate approach to use in studies. In this sense, we think the best way to evaluate response is through changes in composite scores such as DAS, DAS28, SDAI and CDAI and especially using EULAR response criteria, because these are the only measurements to encompass both change in time and endpoint values, assuring that patients with good response have a significant decrease in disease activity and also have reached low disease activity. By using these standard measures of response to treatment, comparison between studies would be facilitated and prediction capacities would be easier to detect.

## Conclusions

In summary, predicting response to MTX and other DMARDs is a stimulating challenge in RA research, not yet fully accomplished. Although it is still not possible to determine whether or not a patient will respond to MTX, we identified clinical and biological factors associated with increased effectiveness: male gender, non-smoking, early disease stage, absence of previous DMARD use, lower baseline disease activity measured by composite scores, concomitant corticosteroids and SE-negativity. Combining distinct factors, adopting new approaches in emerging fields and applying them in larger standardized studies will help define prediction models and reach the longed-for goal of tailor-made therapy.

## Abbreviations

3'-UTR: 3'-untranslated region; 7-OH-MTX: 7-hydroxy-methotrexate; ABC: ATP-binding cassette; ACPA: anti-citrullinated protein antibodies; ACR: American College of Rheumatology; ADA: adenosine deaminase; AICAR: 5-aminoimidazole-4-carbox-amide ribonucleotide; ALT: alanine aminotransferase; AMPd: adenosine monophosphate deaminase; anti-CCP2: second-generation anti-cyclic citrullinated peptide; anti-MCV: anti-modified citrullinated vimentin antibodies; AST: aspartate aminotransferase; ATIC: 5-aminoimidazole-4-carbox-amide ribonucleotide transformylase; bp: base pair; CBC: complete blood count; CDAI: clinical disease activity index; CI: confidence interval; CRP: C reactive protein; DAS: disease activity score; DAS28: disease activity score - 28 joint; DHF: dihydrofolate; DHFR: dihydrofolate reductase; DMARDs: disease-modifying antirheumatic drugs; dTMP: deoxythymidine monophosphate; dUMP: deoxyuridine monophosphate; ESR: erythrocyte sedimentation rate; EULAR: European League Against Rheumatism; FAICAR: 10-formyl 5-aminoimidazole-4-carboxamide ribonucleotide; FPGS: folylpolyglutamate synthetase; GGH: **γ**-glutamyl hydrolase; HAQ: health assessment questionnaire; Hb: hemoglobin; HCQ: hydroxychloroquine; HLA: human leukocyte antigen; HR: hazard ratio; IFN: interferon; IgG: immunoglobulin G; IL: interleukin; IL-1ra: interleukin-1 receptor antagonist; IMP: inosine monophosphate; ITPA: inosine triphosphate pyrophosphatase; Methyl-THF: 5-methyl-tetrahydrofolate; Methylene-THF: 5:10-methylene-tetrahydrofolate; MMP-3: matrix metalloproteinase-3; MS: methionine synthase; MTHFD1: methylene-tetrahydrofolate dehydrogenase; MTHFR: 5:10-methylene-tetrahydrofolate reductase; MTR: 5-methyltetrahydrofolate-homocysteine methyltransferase; MTX: methotrexate; MTX PG: methotrexate polyglutamates; NSAIDs: non-steroidal anti-inflammatory drugs; OR: odds ratio; PBMC: peripheral blood mononuclear cells; RA: rheumatoid arthritis; RBC: red blood cells; RCT: randomized clinical trial; rDAS28: relative disease activity score - 28 joint; RF: rheumatoid factor; RFC1: reduced folate carrier 1; sCD30: soluble CD30; SD: standard deviation; SDAI: simplified disease activity index; SE: shared epitope; SHMT: serine hydroxymethil transferase; sIL-2R: soluble interleukin-2 receptor; SJC: swollen joint count; SNPs: single nucleotide polymorphisms; SR: systematic review; SSZ: sulphasalazine; sTNFR: soluble tumor necrosis factor receptor; THF: tetrahydrofolate; TJC: tender joint count; TNF: tumor necrosis factor; TNFID_50_: dose required to suppress by 50% the production of tumor necrosis factor; TSER: thymidylate synthase enhancer region; TYMS: thymidylate synthase; UA: undifferentiated arthritis; ULN: upper limit of normal; VAS: visual analogue scale.

## Competing interests

The authors declare that they have no competing interests.

## Authors' contributions

All authors participated in the protocol design. VCR performed the literature search, drafted and edited the manuscript, including figures and tables. HC and JEF reviewed, commented and complemented the manuscript. All authors have read and approved the final manuscript.

## Authors' information

VCR, MD is a research trainee at the Rheumatology Research Unit, Instituto de Medicina Molecular, Faculdade de Medicina da Universidade de Lisboa, Lisbon, Portugal. He is also a Rheumatology fellow at the Lisbon Academic Medical Centre, Portugal.

HC, MD, MMSc, PhD is Principal Investigator at the Rheumatology Research Unit, Instituto de Medicina Molecular, Faculdade de Medicina da Universidade de Lisboa. She is Assistant Professor of Rheumatology and a Rheumatology Consultant at the Lisbon Academic Medical Centre. She is also the National Coordinator of Reuma.pt (Rheumatic Diseases Portuguese Register, Portuguese Society of Rheumatology).

JEF, MD, PhD is the Head of the Rheumatology Research Unit and of the Biobank at Instituto de Medicina Molecular, Faculdade de Medicina da Universidade de Lisboa. He is Assistant Professor of Rheumatology and a Rheumatology Consultant at the Lisbon Academic Medical Centre. He is also the President-Elect of the Portuguese Society of Rheumatology.

## Pre-publication history

The pre-publication history for this paper can be accessed here:

http://www.biomedcentral.com/1741-7015/11/17/prepub
